# Protective Effect of Flavonoids from *Ohwia caudata* against Influenza a Virus Infection

**DOI:** 10.3390/molecules25194387

**Published:** 2020-09-24

**Authors:** Eun Bin Kwon, Hye Jin Yang, Jang-Gi Choi, Wei Li

**Affiliations:** Korean Medicine (KM) Application Center, Korea Institute of Oriental Medicine, Daegu 41062, Korea; wrld2931@kiom.re.kr (E.B.K.); hjyang@kiom.re.kr (H.J.Y.); jang-gichoi@kiom.re.kr (J.-G.C.)

**Keywords:** *Ohwia caudata*, flavonoid, influenza A, neuraminidase

## Abstract

To identify new potential anti-influenza compounds, we isolated six flavonoids, 2′-hydroxyl yokovanol (**1**), 2′-hydroxyl neophellamuretin (**2**), yokovanol (**3**), swertisin (**4**), spinosin (**5**), and 7-methyl-apigenin-6-C-β-glucopyranosyl 2″-*O*-β-d-xylopyranoside (**6**) from MeOH extractions of *Ohwia caudata*. We screened these compounds for antiviral activity using green fluorescent protein (GFP)-expressing H1N1 (A/PR/8/34) influenza A-infected RAW 264.7 cells. Compounds **1** and **3** exhibited significant inhibitory effects against influenza A viral infection in co-treatment conditions. In addition, compounds **1** and **3** reduced viral protein levels, including M1, M2, HA, and neuraminidase (NA), and suppressed neuraminidase (NA) activity in RAW 264.7 cells. These findings demonstrated that 2′-hydroxyl yokovanol and yokovanol, isolated from *O. caudate*, inhibit influenza A virus by suppressing NA activity. The moderate inhibitory activities of these flavonoids against influenza A virus suggest that they may be developed as novel anti-influenza drugs in the future.

## 1. Introduction

According to the World Health Organization, the influenza A virus (IVA) infects about 10% of the population worldwide [1]. Influenza A is an acute, infectious respiratory disease with high mortality and epidemic potential. Currently, vaccines and therapeutic agents, including neuraminidase (NA) inhibitors, proton channel protein (M2) inhibitors, and entry inhibitors, are used to prevent or treat influenza [2]. 

NA is a viral enzyme that consists of four identical subunits and is localized to the viral membrane. NA plays an important role in the spread of IVA by assisting in the release of virions by cleaving neuraminic acids and glycoprotein linkages. As a consequence, NA inhibition is an attractive target for anti-influenza studies [2]. 

Amantadine and rimantadine specifically target the influenza A virus through their inhibition of the viral M2 protein, which is a proton channel found only in the influenza A virus [3]. In early 2006, the US Centers for Disease Control and Prevention recommended that the use of amantadine be discontinued, as the number of H3N2 cases with amantadine resistance rapidly increased to 92.3% of all cases in the US [4,5]. Since 2006, amantadine resistance has also been reported throughout the rest of the world. Currently, the M2 inhibitors amantadine and rimantadine are no longer used due to drug resistance [4,5]. In contrast, NA inhibitors have inhibitory effects on both type A and type B influenza viruses and these drugs, including oseltamivir and zanamivir, which are recommended as antiviral agents against influenza infections [3]. However, as with other drugs, NA inhibitors exhibit drug resistance as well as side effects [2]. Therefore, it is essential that anti-influenza A therapeutic agents with minimal side effects and high efficacy be identified. For this purpose, various studies have examined the use of natural materials, including those based on traditional medicines with anti-influenza effects [6].

*Ohwia caudata* is a shrub belonging to the family Fabaceae, which is formerly placed in the genus *Desmodium* (as *Desmodium caudatum*). It has traditionally been used for treating rheumatic backache, diarrhea, icterohepatitis, and colds [7]. In a previous report, the chemical composition of *O. caudata* was examined and found to include flavonoids, triterpenoids, and alkaloids. Previous studies demonstrated that the flavonoids from *O. caudata* exhibited free radical scavenging and anti-amyloid beta (Aβ) aggregation activities [8]. However, the potential inhibitory effects of *O. caudata* on influenza A have not yet been examined.

In this study, we sought to isolate potential antiviral compounds from the leaves and stems of *O. caudate*. Six flavonoids were isolated and their antiviral activity was investigated using influenza A-infected RAW 264.7 cells. In addition, we identified compounds that affected NA inhibition.

## 2. Results and Discussion

### 2.1. Isolation and Structural Elucidation

**Figure 1:**
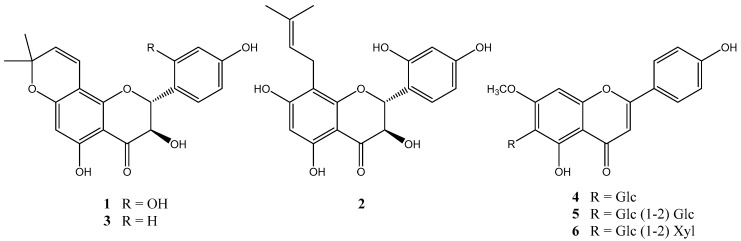
Structure of compounds **1**–**6** from *O. caudata*.

Six flavonoids (**1**–**6**) were isolated from the hexane and n-BuOH soluble fractions of the MeOH extract of *O. caudata* (Figure 1). Their structures were determined by comparing their spectral data with reported literature values and identified as 2′-hydroxyl yokovanol (**1**) [9], 2′-hydroxyl neophellamuretin (**2**) [9], yokovanol (**3**) [10], swertisin (**4**) [11], spinosin (**5**) [12], and 7-methyl-apigenin-6-C-β-glucopyranosyl-2″-*O*-β-d-xylopyranoside (**6**) [13].

### 2.2. Compounds **1** and **3** Inhibit Influenza Vires A (IVA) Infection in RAW 264.7 Cells

**Figure 2:**
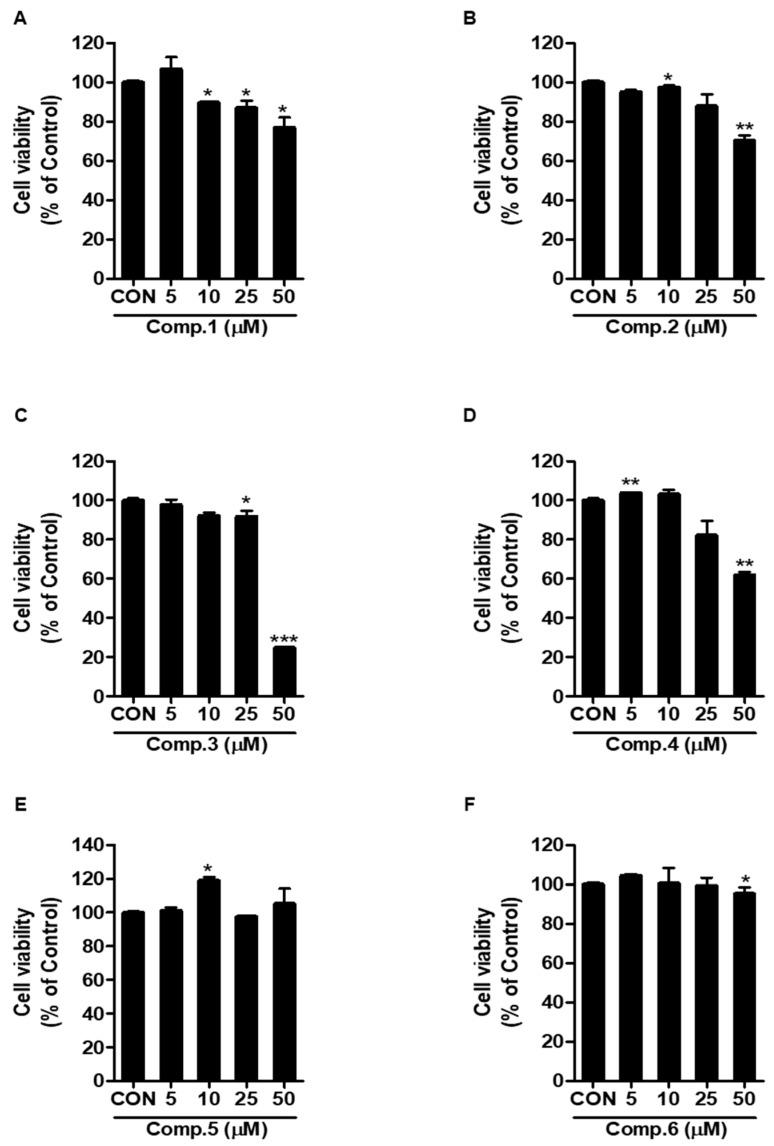
Effect of compounds **1**–**6** on cell viability in Raw 264.7 cells. Cells were incubated with compounds **1**–**6** for 24 h before the 3-(4,5-dimethylthiazol-2-yl)-2,5-diphenyl tetrazolium bromide (MTT) assay. The bar graphs show the mean ± SD of three independent experiments (* *p* < 0.05, ** *p* < 0.01 and *** *p* < 0.001 compared with the dimethyl sulfoxide (DMSO) control).

**Figure 3:**
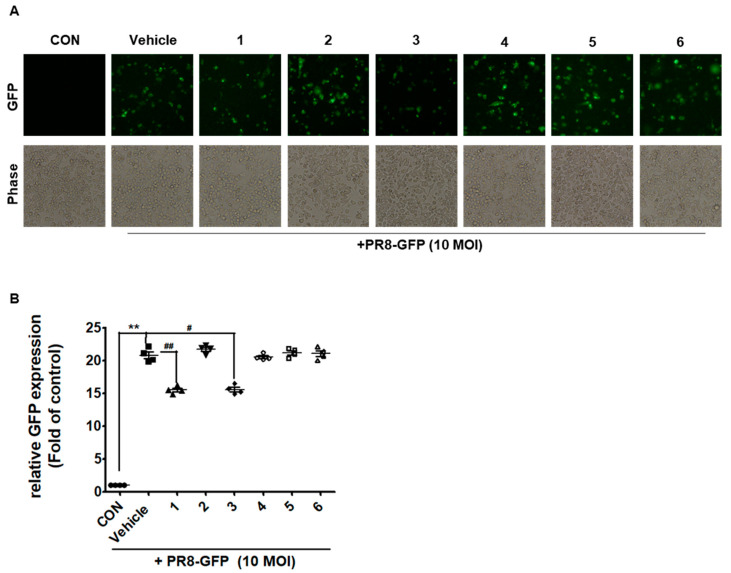
Effect of compounds **1**–**6** on influenza A infection in Raw 264.7 cells. (**A**) Cells were incubated with the mixture (compounds **1**–**6** and 10 MOI PR-GFP) for 24 h and then, images of GFP expression detection by fluorescence microscope. (**B**) Mean fluorescence intensity (MFI), quantitative analysis was performed by flow cytometry. Vehicle (10% DMSO). The bar graphs show the mean ± SD of three independent experiments (** *p* < 0.01 compared with the DMSO control, # *p* < 0.05 and ## *p* < 0.01 compared with the IVA-infected control).

We next evaluated flavonoid compounds **1**–**6** for potential anti-influenza A activity. We first examined the viability of RAW 264.7 cells after treatment with various concentrations of flavonoid compounds **1**–**6**. As shown in Figure 2, flavonoid compounds **1**–**6** did not show cytotoxicity at 5 to 25 μM. Therefore, we used by concentration at 25 μM on screening. Next, RAW 264.7 cells were co-treated with flavonoid compounds **1**–**6** and A/PR/8/34-GFP (10 MOI) IVA. Both compounds **1** and **3** reduced green fluorescent protein (GFP) expression in cells in the co-treatment assay (Figure 3 and Figure S1). However, in pre-treatment and post-treatment assays, flavonoid compounds **1**–**6** did not show any effects (data not shown). These data indicate that compounds **1** and **3** significantly inhibited influenza A viral activity in a co-treatment assay when compared with that of the vehicle. These results suggest that the effect of compounds **1** and **3** in the co-treatment assay could directly affect the virus or prevent the virus from entering the cells.

**Figure 4:**
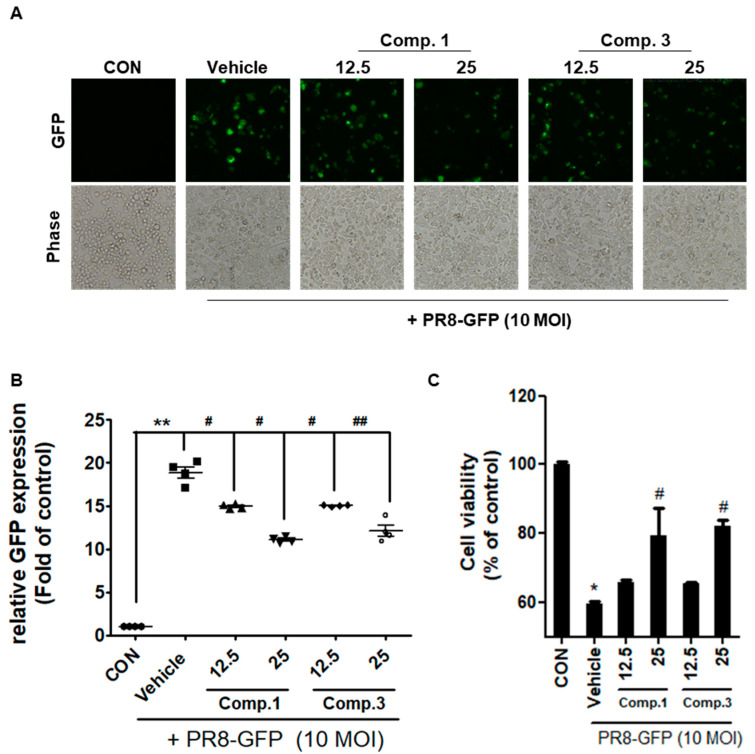
Compounds **1** and **3** inhibits influenza A infection on Raw 264.7 cells. (**A**) Cells were incubated with concentration of compounds **1**, **3**, and 10 MOI PR8-GFP for 24 h and, then, images of GFP expression detection by fluorescence microscope. (**B**) Quantitative analysis was performed by flow cytometry. (**C**) Cell viability was determined 24 h after viral infection by the MTT assay. The bar graphs show the mean ± SD of three independent experiments (* *p* < 0.05 and ** *p* < 0.01 compared with the vehicle (10% DMSO). # *p* < 0.05 and ## *p* < 0.01 compared with the IVA-infected control (vehicle)).

We next examined the expression of A/PR/8/34-GFP virus-induced GFP in RAW 264.7 cells co-treated with multiple concentrations of compounds **1** and **3** (12.5 and 25 μM) and IVA (A/PR/8/34-GFP). Compounds **1** and **3** inhibited virus-induced GFP expression in a dose-dependent manner when compared with that of the vehicle (Figure 4A,B). Consistent with these data, an MTT assay revealed that co-treatment with compounds **1** and **3** suppressed H1N1 virus-induced cell death in RAW 264.7 cells (Figure 4C). These data demonstrate that compounds **1** and **3** can inhibit A/PR/8/34-GFP virus-induced GFP expression and viral cytopathic effect (CPE) when compared with that of the vehicle.

### 2.3. Compounds **1** and **3** Suppress Viral Protein Expression

**Figure 5:**
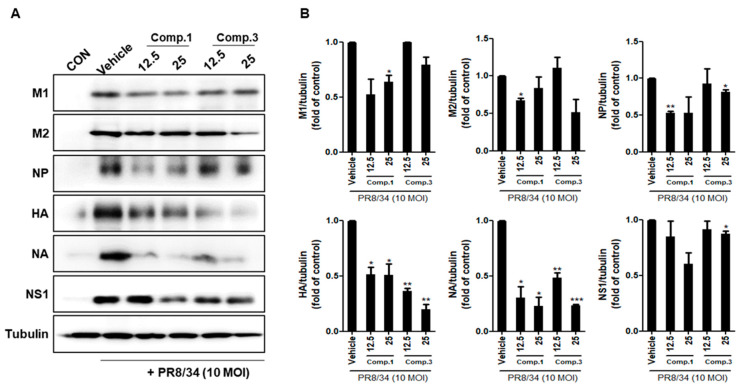
Compounds **1** and **3** suppresses influenza A (A/PR/8/34, H1N1) protein expression. (**A**) Western blot analysis of viral protein expression level was performed after treatment of indicated concentrations of compounds **1** and **3** (12.5 and 25 μM) and 10 MOI H1N1 virus in Raw 264.7 cells. (**B**) Western blot band was quantified by using the Image J software. The bar graphs show the mean ± SD of 3 independent experiments (* *p* < 0.05, ** *p* < 0.01 and *** *p* < 0.001 compared with the vehicle (IVA-infected control)).

**Figure 6:**
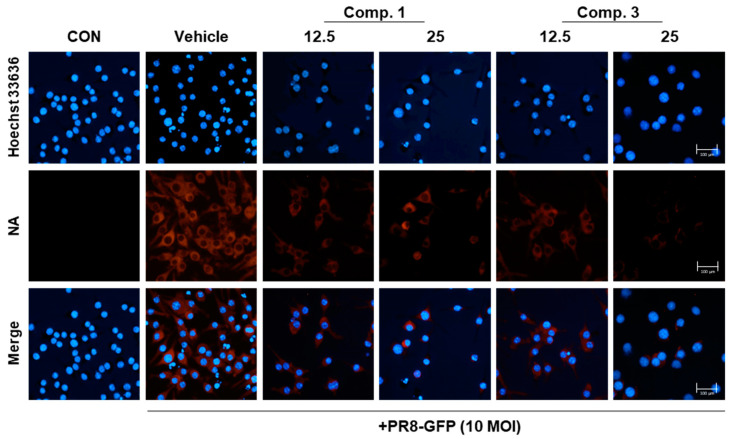
Compounds **1** and **3** reduced NA of expression. Cells were treated with a mixture (compounds **1**, **3**, and 10 MOI PR8-GFP) for 24 h. Cells were examined by fluorescence microscopy after staining with an NA-specific antibody (**Red**). Nuclei were counterstained with Hoechst 33,342 dye (**Blue**).

To determine whether compounds **1** and **3** decreased the expression of viral proteins in IVA-infected RAW 264.7 cells, we performed Western blotting analysis of protein expression. As shown in Figure 5, compounds **1** and **3** suppressed A/PR8/34 viral protein levels (M1, M2, NP, HA, NA, and NS1). In total, 25 μM concentrations of compounds **1** and **3** significantly inhibited NA protein levels by 76.9% and 76.4%, respectively, when compared with the IVA-infected control (vehicle). In addition, we observed a decrease in the expression of NA in a dose-dependent manner in infected RAW 264.7 cells by immunofluorescence analysis (Figure 6). These data suggest that compounds **1** and **3** reduce viral protein levels in IVA-infected RAW 264.7 cells.

### 2.4. Compounds **1** and **3** Reduced NA Activity

**Figure 7:**
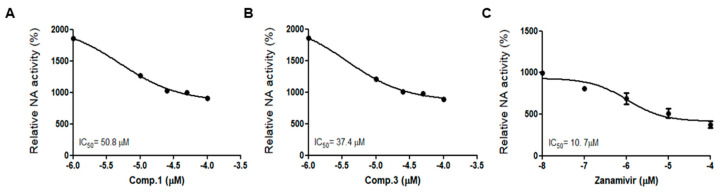
Compounds **1** and **3** inhibited NA activity. A/PR/8/34 was incubated with concentration of compounds **1**, **3**, and 200 mM NA-Fluor substrate was added. After 1 h of incubation at 37 °C, the reaction was terminated by adding NA-Fluor stop solution and monitored via fluorescence spectrometry. (**A**) IC_50_ value of compound 1 on NA activity. (**B**) IC_50_ value of compound 3 on NA activity. (**C**) IC_50_ value of zanamivir on NA activity. Zanamivir used a positive control. The bar graphs show the mean ± SD of three independent experiments.

Next, we investigated the effects of compounds **1** and **3** on NA activity according to previously described materials and methods. Zanamivir, which is an FDA-approved drug for treating influenza, was used as a positive control. The NA activity of H1N1 (P/PR/8/34) was significantly reduced with compounds **1**, **3**, and zanamivir. The results suggest that compounds **1** and **3** has an inhibitory effect on the influenza A virus by inhibiting the NA of H1N1(P/PR/8/34) in a dose-dependent manner compared with that of the vehicle (Figure 7). 

Previous reports shown that flavonoids such as kaempferol, quercetin, and naringenin have anti-influenza effect via inhibited NA activity [14]. However, compounds **1** and **3** isolated from *O. caudate* in this study had not previously been reported to have anti-influenza effects. In this study, we found that compound **1**, 2′-hydroxyl yokovanol, and compound **3**, yokovanol, inhibited the infection of influenza and reduced the NA activity.

In the structure‑activity relationships of six flavonoids (**1**–**6**), compounds **1** and **3** showed the inhibitory effect on NA and viral infection. By comparison of other compounds, a 2,2-dimethyl-2*H* pyran ring located at C-7 and C-8 seems to be a key functional element. Several flavonoids of *O. caudate* were found to possess anti-bacterial, anti-inflammatory, and anti-pyretic activities. However, to our knowledge, the present study is the first to report anti-influenza activity of the chemical components isolated from *O. caudate*.

## 3. Materials and Methods 

### 3.1. General Information

Optical rotations were determined using a Jasco DIP-370 automatic polarimeter. The nuclear magnetic resonance (NMR) spectra were recorded using a JEOL ECA 600 spectrometer (JEOL Ltd., Tokyo, Japan)(^1^H, 600 MHz, ^13^C, 150 MHz), The licence controller qualification (LCQ) advantage trap mass spectrometer (Thermo Finnigan, San Jose, CA, USA) was equipped with an electrospray ionization (ESI) source, and high-resolution electrospray ionization mass spectra (HR-ESI-MS) were obtained using an Agilent 6530 Accurate-Mass quadrupole time-of-flight mass spectrometry (Q-TOF LC/MS) system. Column chromatography was performed using a silica gel (Kieselgel 60, 70–230, and 230–400 mesh, Merck, Darmstadt, Germany), YMC RP-18 resins, and thin layer chromatography (TLC) was performed using pre-coated silica-gel 60 F_254_ and RP-18 F_254_S plates (both 0.25 mm, Merck, Darmstadt, Germany).

### 3.2. Plant Material

The leaves and stems of *O. caudata* were collected in Jeju, Korea, in August 2010 and identified by Prof. Young Ho Kim. A voucher specimen (CNU 10107) was deposited at the Herbarium of College of Pharmacy, Chungnam National University, Korea.

### 3.3. Extraction and Isolation

The dried leaves and stems of *O. caudata* (1.0 kg) were extracted with MeOH under reflux for 9 h (5 L × 3 times) to yield 83.0 g of extract. The extracts were remained for emergency and used for a previous experiment (Li et al. 2014). This extract was suspended in water and partitioned with *n*-hexane to yield 26.0 g of hexane extract and 55.0 g of water extract. The water extract was partitioned with *n*-BuOH to yield 10.5 g of *n*-BuOH extract. Compounds **1** (48.0 mg), **2** (21.0 mg), and **3** (16.0 mg) were isolated from the hexane extract. Compound **4** (55.0 mg), **5** (46.0 mg), and **6** (38.0 mg) were isolated from *n*-BuOH extract.

### 3.4. Cells and Virus

Raw 264.7 cells were maintained in RPMI 1640 supplemented with 10% FBS (Fetal Bovine Serum, Gibco, Thermo Fisher Scientific, Waltham, MA, USA) and 1% antibiotic-anti-myotic (Gibco, Grand Island, NY, USA) at 37 °C, 5% CO_2_ in humidified air. Influenza A (A/PR/8/34) and GFP-tagged A/PR/8/34 (A/PR/8/34-GFP) viruses were used in the previous studies [15]. 

### 3.5. Cell Viability MTT Assay

Cells were seeded in 24-well plates for 24 h and then were treated with various concentrations of flavonoid compounds for 24 h. Cell viability was measured using the MTT assay. Cells were treated with 5 mg/mL of MTT solution for 30 min, and then purple formazan was dissolved in DMSO and the absorbance at 540 nm was measured with a microplate reader (Epoch, BioTek, Irvine, CA, USA). 

### 3.6. Virus Infection and Antiviral Activity Assay

Raw 264.7 cells were cultured in 24-well plates at a density of 1 × 10^5^ cells/well for 18 h. In order to confirm the antiviral effect, three conditions (pre-treatment, co-treatment, and post-treatment) were used for viral infection (10 MOI) and flavonoid compounds **1**–**6** (Figure S2). Influenza virus GFP expression was measured under a fluorescence microscope (Nikon, Tokyo, Japan) and the reduction of the viral infection effect was determined by measuring GFP expression using flow cytometry [16].

### 3.7. Immunofluorescence

IVA-infected Raw 264.7 cells were cultured on cover slips and fixed with 4% paraformaldehyde in PBS for 10 min at room temperature. After washing three times with phosphate-buffered saline (PBS), the fixed cells were permeabilized with 0.1 M glycine for 5 min at room temperature. After three washes with PBS, the cells were incubated with blocking solution (5% BSA in PBS) for 30 min and then with NA antibody (GenoTex, Irvine, CA, USA) overnight at 4 °C for 24 h. Afterward, cells were washed with PBS and then incubated with Alexa568-tagged secondary antibody for 30 min on the rocker. The cells were washed with PBS and Hoechst 33,342 stained for 15 min. After washing, the cover slips were mounted on a slide using mounting media. Cells were visualized with a fluorescence microscope (Lionheart FX automated microscope, BioTek, Irvine, CA, USA).

### 3.8. Western Blots

IVA-infected Raw 264.7 cells were lysed with PRO-PREP protein extraction solution containing protease inhibitor and phosphatase inhibitor on ice for 30 min. After centrifugation at 13,200× *g* for 30 min at 4 °C, the supernatant was collected, and the protein concentration was quantified by the Bradford method [17]. Equal amounts of protein samples were separated by sodium dodecyl sulfate–polyacrylamide gel electrophoresis (SDS-PAGE, 8% to 15% gel) and transferred to polyvinylidene fluoride or polyvinylidene difluoride (PVDF) membranes. After being blocked with Ez-Block Chemi (Amherst, MA, USA), blots were incubated with primary anti-M1, -M2, -NP, -HA, -NA, -NS1(GenoTex, Irvine, CA, USA), and -tubulin antibodies (1:1000 dilution). Primary antibodies were washed three times with wash buffer (TBS-T) and incubated with horseradish peroxidase (HRP)-conjugated secondary antibodies (1:5000 dilution) at room temperature and the detection ChemiDoc imaging system (UVITEC, Cleaver scientific Ltd., Warwickshire, UK) with an enhanced chemiluminescence reagent (Thermo Scientific, Rockford, IL, USA). Densitometry quantification was performed using NIH Image J program (National Institutes of Health, Bethesda, MD, USA).

### 3.9. NA Activity Assay 

NA activity was determined using the NA-Flour^TM^ Influenza Neuraminidase Assay Kit (Applied Biosystems, Foster City, CA, USA), according to the manufacturer’s instructions. Concentrations of compounds **1** and **3** were added in 96 white well-plates. In addition, it was then incubated with 200 μM NA-Fluor™ Substrate at 37 °C for 1 h. Terminate the reaction by adding 100 μL of NA-Fluor™ Stop Solution to each well and then detect it by GloMaX EXPLORER (Promega, Madison, WI, USA).

### 3.10. Statistical Analysis

Data are presented as mean ± standard deviation (SD). Statistical analysis was performed using Student’s t-test for the in vitro experiments. Differences were considered significant at *p* < 0.05 (*), *p* < 0.01 (**), and *p* < 0.001 (***).

## 4. Conclusions

This study is the first to demonstrate the protective effect of flavonoids derived from *Ohwia caudate* against type A influenza virus infection and to propose use as an alternative to conventional anti-influenza therapy. We identified 2′-hydroxyl yokovanol (**1**) and yokovanol (**3**) as active ingredients of *O. caudata* that inhibit NA activity. These compounds may be useful as a therapeutic agent or prophylactic agents to limit viral infection through NA inhibition.

## Supplementary Materials

The following are available online. Figure S1: Compounds **1** and **3** inhibit influenza A infection in MDCK cells and Figure S2: Schematic diagram of the anti-influenza A virus assay is available online.
