# Supplementary material for: Protective Effect of Flavonoids from Ohwia caudata against Influenza a Virus Infection
Source: Molecules. 2020 Sep 24;25(19):4387. doi: 10.3390/molecules25194387 (PMC7583851; doi:10.3390/molecules25194387)
Supplement: Supplementary file 1 [file molecules-25-04387-s001.pdf]

Supplementary Data

Protective effect of flavonoids from *Ohwia caudata* against influenza A virus infection

Eun Bin Kwon [1], Hye Jin Yang [1], Jang-Gi Choi [1] and Wei Li [1,*]

[1] Korean Medicine (KM) Application Center, Korea Institute of Oriental Medicine, Daegu 41062, Republic of Korea; wrld2931@kiom.re.kr (E.B.K,); hjyang@kiom.re.kr (H.J.Y.); jang-gichoi@kiom.re.kr (J.H.C.); liwei1986@kiom.re.kr (W.L.)

*Correspondence: liwei1986@kiom.re.kr; Tel.: +82-53-940-3874 (W.L.)

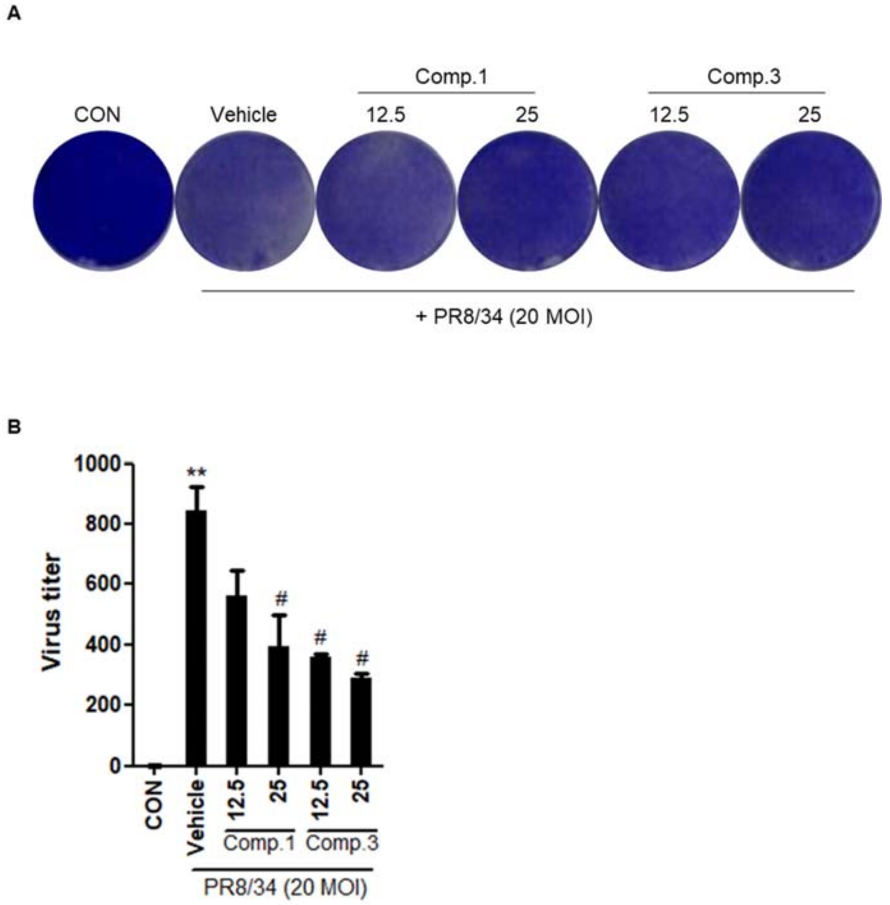

Figure S1. Compounds 1 and 3 inhibit influenza A infection in MDCK cells. The Cells were co-treated with compound 1 and 3(12.5 and 25 μM) and 20 MOI influenza A (A/P/PR8/34) at 4°C for 2 h and then incubation at 37°C for 2 h. After reaction, remove mixture and then incubated with media containing 10% FBS at 37°C for 3day before plaque assay. (A) Image for plaque assay in MDCK (B) Quantitative analysis was performed by microscopy. The bar graphs show the mean ± SD of 3 independent experiments (∗∗p < 0.01 compared with the DMSO control; #p < 0.05 compared with the IVA-infected control).

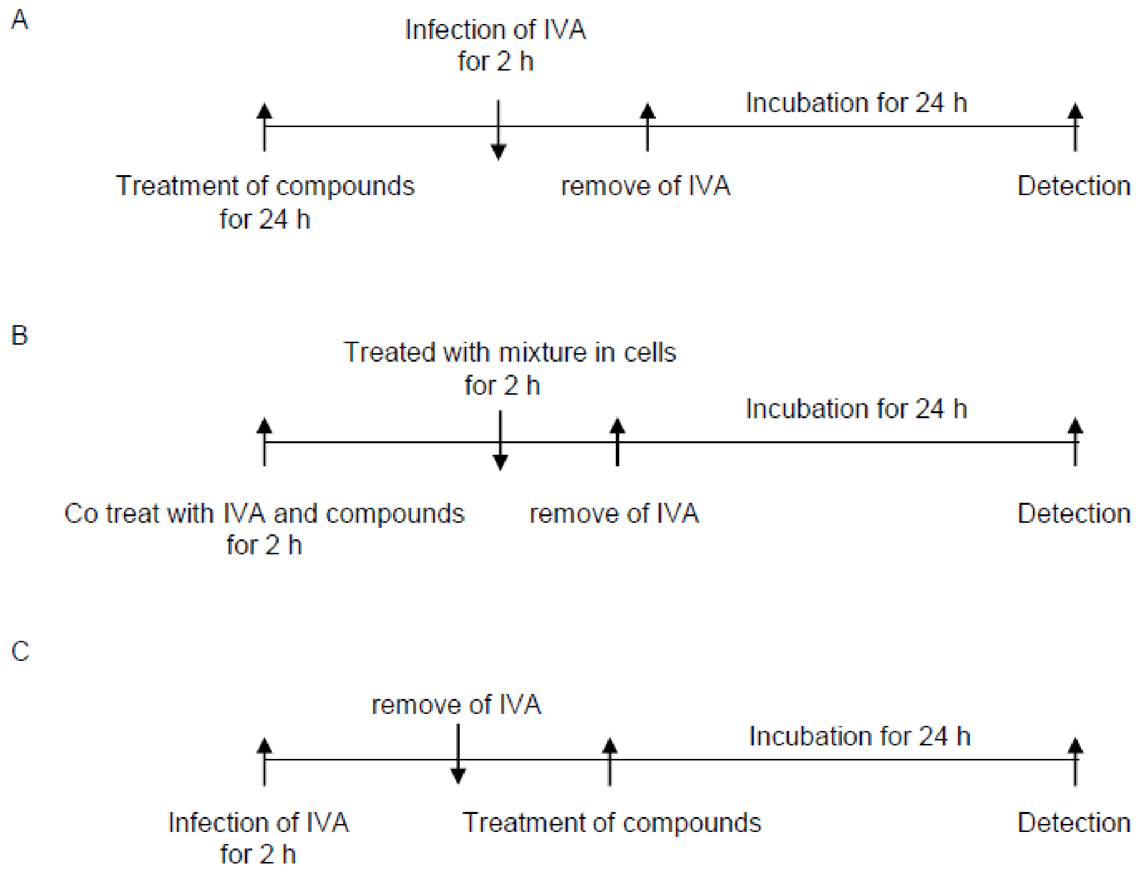

Figure S2. Schematic diagram of the anti-influenza A virus assay. (A) pre-treatment assay (B) co-treatment assay (C) post-treatment assay.
